# Indents

**Published:** 1913-09-15

**Authors:** 


### INDENTS.

**☞Brief Articles of Technical or Scientific Value will receive notice and comment. Contributions invited.**

*Close-Plated Platinum Utensils.* Patents have been taken out by a New York inventor for close-plated platinum articles to take the place of platinum dishes, crucibles and similar apparatus. The process consists of welding platinum to a base of steel or nickel and then rolling into sheet. A platinum-coated steel or nickel is thus obtained. This is then spun into the desired shapes. The surface is, of course, coated with the platinum, but the edges are exposed base metal. To cover these up, platinum is fused on the edges and a vessel or article is obtained completely coated with platinum, but at a much less cost than a solid platinum one could be made. The interior of steel or nickel can, of course, be made of sufficient thickness to render the articles as rigid as desired. *Dental Scrap Book.*

*Justice for Jim Jam Jems.* This publication recently made a viscious attack upon the Secretary of the American Medical Association and Dr. W. A. Abbot Editor of Clinical Medica.—S. H. Clark and C. H. Crockard of Bismarck, North Dakota, edit and publish *Jim Jam Jems,* a sheet that has printed not only obscene and indecent matter but also personal attacks on numerous individuals and organizations, including the American Medical Association and some of its officers and members. To the interests it represents it has given value received. The publication has not been distributed through the mails, having as we understand, been debarred from the use of the United States postal service; the express companies have been utilized instead. It may interest our readers to know that the editor and publisher have recently been found guilty of sending obscene matter in interstate commerce. Each has been sentenced by a United States judge to four years' imprisonment in the federal penitentiary and to pay a fine of $2,000 and half the costs of the prosecution. Incidentally, the daily press has within the past few days reported the conviction of a newsdealer for handling this obscene sheet. The papers state that this in the first of a number of suits the government has started against other newsdealers.—*A. M. A. Journal.*

*Property Versus Life.*

To the legal mind, apparently, the rights of property have always seemed of more importance than human life. For hundreds of years it was possible for a man brutally to maltreat his child with less legal risk than if he hand poached a hare. Gradually human life became more valuable; but even to-day it fails to receive the protection that is accorded to property. It is no uncommon thing to find in British newspapers cases in which a drunken navvy has kicked and otherwise abused his wife, to receive no greater punishment at the hands of the law than a paltry fine; while the unhappy wight who, driven by hunger, steals a loaf, is sent to prison, Nor do we need to go oversea to find such instances of the worship of property. An excellent illustration of the workings of the legal mind in problems of this kind is to be found in a study of ten Notices of Judgments issued by the United States Department of Agriculture and giving in detail the account of ten violations of the Food and Drugs Act. These ten cases deal with charges brought against the firm of Hawley and Hoops, New York, who are in the candy business. Hawley and Hoops sell what is known as "penny goods;" that is, the kind of candy purchased by the little tot who has been given a penny to spend. Ten different specimens of Hawley and Hoops' penny goods were seized by the officials of the Bureau of Chemistry and analyzed. All of them were found to be adulterated with arsenic and most of them contained shellac. All of them were being sold as chocolate candies yet the officers reported that some did not even have the predominating flavor of chocolate. In every case the firm pleaded guilty. In nine out of the ten cases no penalty was imposed, the court suspending judgment. In the tenth case a fine of $50 was imposed. The case in which a fine was imposed was the one, and the only one in which the company had not merely sold poisonous product to little children but had misstated the net weight of the package in which the arsenic-containing candies came! Selling to little children as chocolate candies a mixture containing arsenic and shellac but not containing even the predominating flavor of chocolate is, apparently, in the eyes of the law, a trivial offense. But selling to a dealer a package marked five pounds that really contained only four pounds fourteen and five-eighths ounces, that is a crime!—*Journal, A. M. A.*

*The Cure of Mouth Breathing.* The procedure now usually followed in establishing nasal respiration in cases of mouth breathing consists in carrying out operative measures, whenever these are indicated, prescribing breathing exercises, and requiring the patient voluntarily to respire through the normal channels. In some instances, as is well known, mouth breathing persists notwithstanding these measures, especially at night, when volition is in abeyance. Unsatisfactory trial of strapping or bandaging at night in these cases has led W. W. James (*Proceedings of the Royal Society of Medicine*, May, 1913) to devise an apparatus consisting of a wire frame of German silver, gilded after completion, over which thin sheet rubber is stretched. The apparatus is placed at night inside the lips and cheeks, resting upon the outer surfaces of the teeth and gums, and renders mouth breathing quite impossible. An accurate model of the jaws, extending as far back as the first molars on either side, is essential if the wire frame is to be a success. The frame consists of two horizontal wires—the upper dipping down in the midline to form a depression corresponding with the frenum labii, and five vertical wires, all evenly soldered together. Rubbing of the mucous membranes is avoided by making the frame large enough to be steady and by avoiding bony eminences. A supply of rubber is given to the patient, with directions to change it frequently.

Such conditions as gingivitis and pyorrhea alveolaris are markedly improved, according to James ,by the use of this apparatus. Chronic sore throat and nasal catarrh are also sometimes greatly benefited. The apparatus should be worn until normal resp'ration is quite established, when it may be left off on alternate nights and later completely.—*Pacific Gazette.*

*Mercuric Chlorid in Surgery.*

Marquis ascribes to Koch the reputation enjoyed by mercuric chlorid as the "sublime antiseptic." He reviews its history and reports experimental and clinical research, the results of which he says, have pricked the bubble of its reputation and shown it to be the least penetrating of the surgical disinfectants in vogue and, in spite of its great antiseptic power, to be the least bactericidal of all. —*Journal, A. M. A.*

*The Antiquity of Bridge Work*

While the origin of dentistry is shrouded in the mists of antiquity and the historical records of that time, are to say the least obscure, one can nevertheless trace the origin of bridge work without undue stretch of the imagination to a period between 450 and 400 B. C. Herodotus of Halicarnassus, the Egyptian historian, is probably the first of the ancient writers to speak of medical specialties and specialists, and includes in the group those physicians who devoted themselves to the treatment of diseases of the teeth. The Egyptian and Grecian dentists of that period were more in the line of dental physicians while the Romans and Phoenicians of the same period were not only dental therapeutists but also dental prosthetists. We have it on the authority of such dental historians as Guerini and Lemerle that between the fifth and fourth centuries before the christian era loose teeth were held together by means of strands of gold wire and that in those cases in which the anterior teeth had been lost either through disease or accident they were replaced by appliances consisting of crude imitations of the lost organs carved out of the teeth of animals, bound together by means of thin bands of soft gold and held *in situ* by tying the appliance to the neighboring sound teeth. Five of these early Etruscan appliances were found in or near the necropolis of Lucius Tarquinius Superbus and date back to about 496 B. C. or thereabouts. These appliances of unique historical value are now housed, two in the Civic Museum of Cornetto, two in the Museum of Count Bruschi, and one in the Museum of Pope Julius in Rome. These prosthetic pieces were made the subject of a communication to the Eleventh International Congress of Medicine and Hygiene, held in 1894, by Professor Guerini, the dental historian, and were therein minutely described. In Professor Lemerle's work, "Notice Sur L'Historie de l'Art Dentaire," Paris, 1900, etchings reproducing these appliances are to be found at page 9 of the book, and from them one can obtain a correct conception of the status of dental mechanics at that period of the world's history.

If a parallel be drawn between mechanical dentistry in the fourth or fifth century B. C., and the present day status of this branch of the art of dentistry, judged from the viewpoint of the technique required to produce the appliances under discussion, one is forced to the conclusion that after all the dictum that Rome was not built in one day, is applicable in all of its metaphoric significance to this branch of prosthetic dentistry. It has required nearly 2400 years of effort to bring about that relative degree of perfection in this branch of prosthetic dentistry in which the American genius of today so conspicuously excells.—Dr. Endelman, in *Pacific Gazette.*

*Malposed Teeth as Bridge Supports.* Take, if you will, a case where the lower second bicuspid and first molars are missing and the second molar has leaned forward into the space. The second molar has, from its movement forward, raised up at its distoocclusо-proximal angle, and been unmercifully hammered into the first stages of pyorrhea; has it no chance for a few years of usefulness? I think it has.

I would suggest this test: If, upon pushing the molar in question firmly back against its posterior socket and holding it steady, the tooth does not permit of perpendicular movement, (up and down in its sockets), and is fairly rigid against lateral movement it is possible to make it a useful member of the dental arch. Of course it is only fair to your patient and yourself to state the case fully and fairly, and if they, realize ing the uncertainty or lack of possible permanency, are still desirous of giving it a trial (you will be surpriesd how many patients, dreading the loss of teeth and the wearing of removable substitutes, are willing), I would suggest the following:

Band accurately the two anterior teeth of the space and solder the two bands together before setting the now twin bands with cement, attach to the loosened molar an anchor band slightly crimped at its gingival borders to give it greater gripping power; fastening between the anterior bands and the molar anchor band a jackscrew, which can be gradually turned up until the molar has been driven back firmly into its sockets, when it can be left for six months before removing the fixture. If the molar has become firm, you may now proceed with the bridge.—E. R. Carpenter, in *Review*.

*Moving Mature Teeth.*

The slight but forcible movement of teeth under the stress of mastication. I consider it important that the occlusal surfaces of the bridge be so adjusted that the teeth will exert as little lateral stress as possible. The seating of the inlays should be firm and strong and forces of mastication be brought evenly and squarely upon the bridge so as to drive them to place rather than out of place. Any lateral stress caused by the teeth striking on one side or the other of a sraight line between the piers, or by striking a diagonal surface with a shearing movement, must soon loosen the inlay. In my opinion the various forces of mastication should be considered the prime influence of failure to be skilfully guarded against by mechanical construction and adjustment of occlusal surfaces.

In regard to the movement of loosened pyorrhea teeth, or of teeth that have drifted into an inclined position, and which you wish to straighten up, to use for abutments, there is no possible objection or difficulty in moving them, providing the patient is not too old. One of the surest and most satisfactory ways to make firm attachments to the teeth or roots to be moved, by cemented bands if possible, to which proper attachments are soldered for the application of a positive screw force. The importance of this kind of force in cases of this character especially, is the advantage of being able to regulate the magnitude and frequency of application. A kind of apparatus also which sustains the tooth firmly in its socket while it is being moved in physiological periods of work and rest, a method of movement moreover. which is not possible with any kind of continuous spring force. The difference between these two kinds of forces for the movement of teeth, is not so important for young patients, but after adolescence, and especially in sluggish or diseased activities of the peridental membranes the sustained positive method of movement must appeal to everyone who considers its advantages.

As pointed out in a paper which I read before this society several years ago, the rigidity of the grasp and positive application of force with alternate periods of rest, is particularly applicable for the movement of pyorrhea teeth. If the calculi is first carefully and thoroughly removed, I feel assured from many cases in my own practice that the peculiar stimulation of dormant activities which arise from this method of movement renders it one of the most potent of all the factors of treatment in restoring the diseased tissues to health, and which in some instances has resulted in a surprising proliferation and restoration of alveolar tissue.—Dr. C. S. Case, in *Dental Review*.

*The Importance of Mixing Cement.* I do not suppose there is any metal spatula which will not become corroded by mixing cement. German silver can be affected by abrasion. The main objection is the discoloration. The tantalum spatula which has been advocated is proof against chemical action and is a shade harder than some other metals, but a mix with a tantalum spatula cannot be made without showing discoloration. You can start with a white cement and come out with a dark gray one when mixing with a tantalum spatula. I prefer the heavy glass slab to a thin piece. If the thin glass plate is not held directly in the fingers there would not be much change of temperature imparted by the hand. In any case, when the glass slab becomes scratched, throw it away. If you are using window glass you can throw it away with a better heart than if you paid $1.50 for it. This question of temperature of the slab impresses me as being of greater importance the more I see of the difficulties experienced by dentists in cement mixing. To become master of the situation under all temperatures and atmospheric conditions, I believe that the progressive and careful dentist will, in time, come to be governed by the readings of a hygrometer in his operating room and use some form of mixing slab which embodies a thermometer and be careful to have that thermometer indicate a definite temperature.—W. V-B. Ames, in *Review.*

*Is There Metabolism in Human Dentin?* The question whether any metabolic changes take place in human dentin after the tooth is completely formed has for a long time been discussed, and opinions are still greatly divided. Charles Tomes, in his "Manual of Dental Anatomy," says: "In the hard or unvascular dentin, some degree of nutrition is perhaps provided for by the penetration of the whole thickness of the tissue by protoplasmic fibers, the dentinal fibers." Other authorities speak as vaguely about this subject. While some investigators, particularly in America, deny the presence of any metabolism in the completely formed dentin, others as decidedly assume its presence, without giving any conclusive proofs of their hypothesis. The following case that come to my knowledge, proves, in my opinion, that a very active metabolism may take place in dentin:

Mr. K , 52 years of age, a healthy man of a strong constitution, slipped two years ago in the street and fell headlong on his face, so that his chin and nose were bruised and bleeding. The four upper incisors were very painful and a little loose. On closer inspection, a few days after the accident, the central incisors displayed reddish spots, which covered almost half of the labial surface and were darkest in the center. The right lateral incisor exhibited the same aspect, only the red spot was smaller. I advised the patient to have the pulps of these four teeth removed and the teeth bleached and properly treated, warning him that the pulps of these four teeth would die, which would give rise to further complications, while the teeth themselves would become still darker. The patient feared that the proposed treatment would cause him great pain, and refused to submit to it, and on his own account painted his gums with tincture of iodin.

My prognosis was partly realized afterward. For some weeks the patient had severe pain in those four incisors, which finally wore off. The reddish spots on the two central and the right lateral incisors had become dark blue, and had almost the color of a so-called blood-blister. Once more I advised the patient to have these teeth properly treated, but without any success. For some time, I did not hear any more about this case.

The patient later communicated to me that those discolored teeth had become white again, which I at first would not believe, but a single look at his teeth convinced me that his assurance was true. On my request, the patient allowed me to ascertain by the aid of the electric current that the pulps of the affected teeth had retained their full vitality.

I also noticed that the spots which had been dark blue were still faintly distinguished from the rest of the tooth by a yellowish, hardly perceptible tint.

Now, four years later, the sound teeth are still in the same good condition, and the pulps react normally.

It is evident that we have here an extravasation of blood which had penetrated the dentin, was decomposed, and almost entirely resorbed again after some time. In my opinion this case is not to be explained in any other way than by acknowledging the presence of a fairly active metabolism in the dentin.—Alex Mertens, in Aug. *Cosmos*.

